# The complete genome sequence of *Stevia rebaudiana*, the Sweetleaf

**DOI:** 10.12688/f1000research.24396.1

**Published:** 2020-07-21

**Authors:** Kathleen O'Neill, Stacy Pirro

**Affiliations:** 1 IRIDIAN GENOMES, Bethesda, MD, 20817, USA

**Keywords:** Stevia rebaudiana, Sweetleaf, genome, assembly, annotation

## Abstract

The Sweetleaf ( *Stevia rebaudiana*: Asteraceae) is widely grown for use as a sweetener.  We present the whole genome sequence and annotation of this species.  A total of 146,838,888 paired-end reads consisting of 22.2G bases were obtained by sequencing one leaf from a commercially grown seedling.  The reads were assembled by a de-novo method followed by alignment to related species.   Annotation was performed via GenMark-ES. The raw and assembled data is publicly available via GenBank: Sequence Read Archive ( SRR6792730) and Assembly ( GCA_009936405).

## Introduction

The Sweetleaf ( *Stevia rebaudiana*: Asteraceae) is cultivated commercially for use as a sweetener. The sweetness is due to various steviol glycosides, primarily stevioside and rebaudioside. These compounds have 200-300X the sweetness of sugar ( Abdullateef & Osman, 2012) but have no calories. The market for raw Stevia and derived products is expected to exceed 1B USD by 2021 ( International Stevia Council, 2017).

*Stevia rebaudiana* has been used as a sweetener for centuries in Brazil and Paraguay ( Misra *et al.*, 2011). Botanist Moisés Santiago Bertoni first described the plant as growing in eastern Paraguay and noted its use as a sweetener ( Bertoni, 1899).

Chemists Bridel and Lavielle isolated the glycosides stevioside and rebaudioside that give the leaves their sweet taste ( Bridel & Lavielle, 1931). The chemical structures of the aglycone steviol and its glycoside have been solved ( Mosettig & Nes, 1955).

A complete genome sequence for this species will assist with discovering markers for crop yields, disease and drought resistance, and determining the biochemical pathways for the relevant metabolites.

## Methods

A single commercially grown *Stevia rebaudiana* plant was used for this study (Behnke Nurseries, Beltsville, MD, USA). DNA extraction was performed on tissue from a single leaf using the Qiagen DNAeasy genomic extraction kit for plants, using the standard process. A paired-end sequencing library was constructed using the Illumina TruSeq kit, according to the manufacturer’s instructions. The library was sequenced on an Illumina Hi-Seq platform in paired-end, 2 × 150bp format.

The resulting fastq files were trimmed of adapter/primer sequence and low-quality regions with Trimmomatic v0.33 ( Bolger *et al.*, 2014). The trimmed sequence was assembled by SPAdes v2.5 ( Bankevich *et al.*, 2012) followed by a finishing step using RagTag v1.0.0 ( Alonge, 2020) to make additional contig joins based on conserved regions in related plant species: *Erigeron canadensis* ( GCA_010389155), *Mikania micrantha* ( GCA_009363875), and *Helianthus annuus* ( GCA_002127325). Default parameters were used for all assembly steps.

Annotation was performed using GeneMark-ES v2.0 ( Lomsadze *et al.*, 2005). Annotation was performed fully de novo without a curated training set and default parameters.

## Results

The genome assembly yielded a total sequence length of 411,383,069 bp over 55,557 scaffolds with an N50 of 37,276,437. The GeneMark-ES annotation resulted in 24,994 genes.

## Data availability

### Underlying data

Raw and assembled data is publicly available via GenBank:

Raw genome of *Stevia rebaudiana*, Accession number SRR6792730: https://www.ncbi.nlm.nih.gov/sra/?term=SRR6792730

Assembly of *Stevia rebaudiana*, Accession number ASM993640v1: https://www.ncbi.nlm.nih.gov/assembly/GCA_009936405.1/
