# Supplementary material for: The complete genome sequence of Stevia rebaudiana, the Sweetleaf
Source: F1000Res. 2020 Jul 21;9:751. [Version 1] doi: 10.12688/f1000research.24396.1 (PMC7676390; doi:10.12688/f1000research.24396.1)
Supplement: Peer review history

# Peer review: Reviewer response for version 1

**DOI:** 10.5256/f1000research.26914.r74315

**Published:** 2020-11-18

**Authors:** Dawson White

**Affiliations:** 1 Department of Science and Education, Field Museum of Natural History, Chicago, IL, USA

Stevia is an economically important plant with no prior genomic resource for researchers. Standard methods were used for laboratory preparation, sequencing, assembly, and annotation of the genome. The resulting assembly and gene annotations are good based on the provided statistics.

 I would like to see that a voucher specimen of the sequenced plant (or a similar individual) at Behnke Nursuries was deposited in a herbarium. This is important for reproducible science and its omission almost causes me to mark that the methods are only "partly" sufficient.

Are sufficient details of methods and materials provided to allow replication by others?

Yes

Is the rationale for creating the dataset(s) clearly described?

Yes

Are the datasets clearly presented in a useable and accessible format?

Yes

Are the protocols appropriate and is the work technically sound?

Yes

Reviewer Expertise:

Plant systematics.

I confirm that I have read this submission and believe that I have an appropriate level of expertise to confirm that it is of an acceptable scientific standard.

# Peer review: Reviewer response for version 1

**DOI:** 10.5256/f1000research.26914.r67544

**Published:** 2020-08-07

**Authors:** Andrew Miller

**Affiliations:** 1 University of Illinois, Illinois, USA; 2 Illinois Natural History Survey, Illinois, USA

The authors present the whole genome sequence of Sweetleaf, the plant where the sweetener, Stevia, is derived.  I am surprised someone has not already sequenced this genome - it is certainly timely.  The data are well presented and publicly available through NCBI.  It is well-written and worthy of indexing. I only have minor comments below:

1. Should the keywords repeat words found in the title?
2. Can more be said in the Results about the genome?

Are sufficient details of methods and materials provided to allow replication by others?

Yes

Is the rationale for creating the dataset(s) clearly described?

Yes

Are the datasets clearly presented in a useable and accessible format?

Yes

Are the protocols appropriate and is the work technically sound?

Yes

Reviewer Expertise:

Sanger sequencing; mycology

I confirm that I have read this submission and believe that I have an appropriate level of expertise to confirm that it is of an acceptable scientific standard.

# Peer review: Reviewer response for version 1

**DOI:** 10.5256/f1000research.26914.r68815

**Published:** 2020-08-07

**Authors:** Eric Lu Zhang

**Affiliations:** 1 Department of Computer Science, Hong Kong Baptist University, Kowloon Tong, Hong Kong

The authors sequenced and assembled the genome of Sweetleaf by Illumina short-reads and annotated the genes using de novo prediction. The data could be useful to the filed, but some points need to be clarified:

1. ​​​​​​Commonly, the first step of genome assembly is to estimate the genome size using k-mer frequency distribution. The authors should add such analysis in the revision. 
2. Based on the results in (1), the genome size of Stevia rebaudiana is ca. 1.3Gb. I don't think SPAdes is a good choice as it only works well on the small geneomes.
3. Gene prediction is too simple and may loose considerable genes. The prediction should include multifaceted information, such as de novo prediction, Homology-based genes prediction *et al.*
4. Add a citation for "standard process" of DNA extraction and the model of sequencing platform, e.g. Hi-Seq 2500.
5. It is better to include a table to show the statistics, such as N50, genome size, the number of genes.
6. Both of the assemblies from short-reads and short-reads+RagTag should be provided, becasue RagTag may introduce certain bias.

Are sufficient details of methods and materials provided to allow replication by others?

Partly

Is the rationale for creating the dataset(s) clearly described?

Yes

Are the datasets clearly presented in a useable and accessible format?

Yes

Are the protocols appropriate and is the work technically sound?

Partly

Reviewer Expertise:

Computational genomics, bioinformatics

I confirm that I have read this submission and believe that I have an appropriate level of expertise to confirm that it is of an acceptable scientific standard, however I have significant reservations, as outlined above.
